# Stress-induced changes in miRNA biogenesis and functioning

**DOI:** 10.1007/s00018-017-2591-0

**Published:** 2017-07-17

**Authors:** Marta Olejniczak, Anna Kotowska-Zimmer, Wlodzimierz Krzyzosiak

**Affiliations:** Department of Molecular Biomedicine, Institute of Bioorganic Chemistry, Polish Academy of Sciences, Noskowskiego 12/14, 61-704 Poznan, Poland

**Keywords:** Inflammation, Neurodegeneration, Drosha, Alzheimer’s disease, Parkinson’s disease, Huntington’s disease, ALS

## Abstract

MicroRNAs (miRNAs) are small, noncoding RNAs that play key roles in the regulation of cellular homeostasis in eukaryotic organisms. There is emerging evidence that some of these processes are influenced by various forms of cellular stresses, including DNA damage, pathogen invasion or chronic stress associated with diseases. Many reports over the last decade demonstrate examples of stress-induced miRNA deregulation at the level of transcription, processing, subcellular localization and functioning. Moreover, core miRNA biogenesis proteins and their interactions with partners can be selectively regulated in response to stress signaling. However, little is known about the role of isomiRs and the interactions of miRNA with non-canonical targets in the context of the stress response. In this review, we summarize the current knowledge on miRNA functions under various stresses, including chronic stress and miRNA deregulation in the pathogenesis of age-associated neurodegenerative disorders.

**Electronic supplementary material:**

The online version of this article (doi:10.1007/s00018-017-2591-0) contains supplementary material, which is available to authorized users.

## Introduction

Cellular stress can be defined as changes in the environment that significantly disturb cellular homeostasis and cause damage to macromolecules such as proteins, DNA, RNA and lipids. Cells respond to stress by activating mechanisms leading to the restoration of cellular homeostasis or adaptation to environmental conditions through growth arrest, repair or clearance of damaged macromolecules and changes in the gene expression programs. Moreover, excessive damage resulting from the high dose, time of exposition or potency of a stressor may trigger programmed cell death (apoptosis). There is growing evidence to confirm the critical role of non-coding RNAs, in particular miRNAs in cellular stress responses [1–4]. These short RNAs, approximately 22 nt in length, control the expression of more than half of the protein-coding genes responsible for major cellular processes such as proliferation and cell cycle progression, differentiation, immune response and apoptosis [5]. miRNAs, post-transcriptional regulators of gene expression, offer the possibility of fast and economical regulation during stress by targeting multiple transcripts at the same time and without the need to synthesize proteins.

**Fig. 1:**
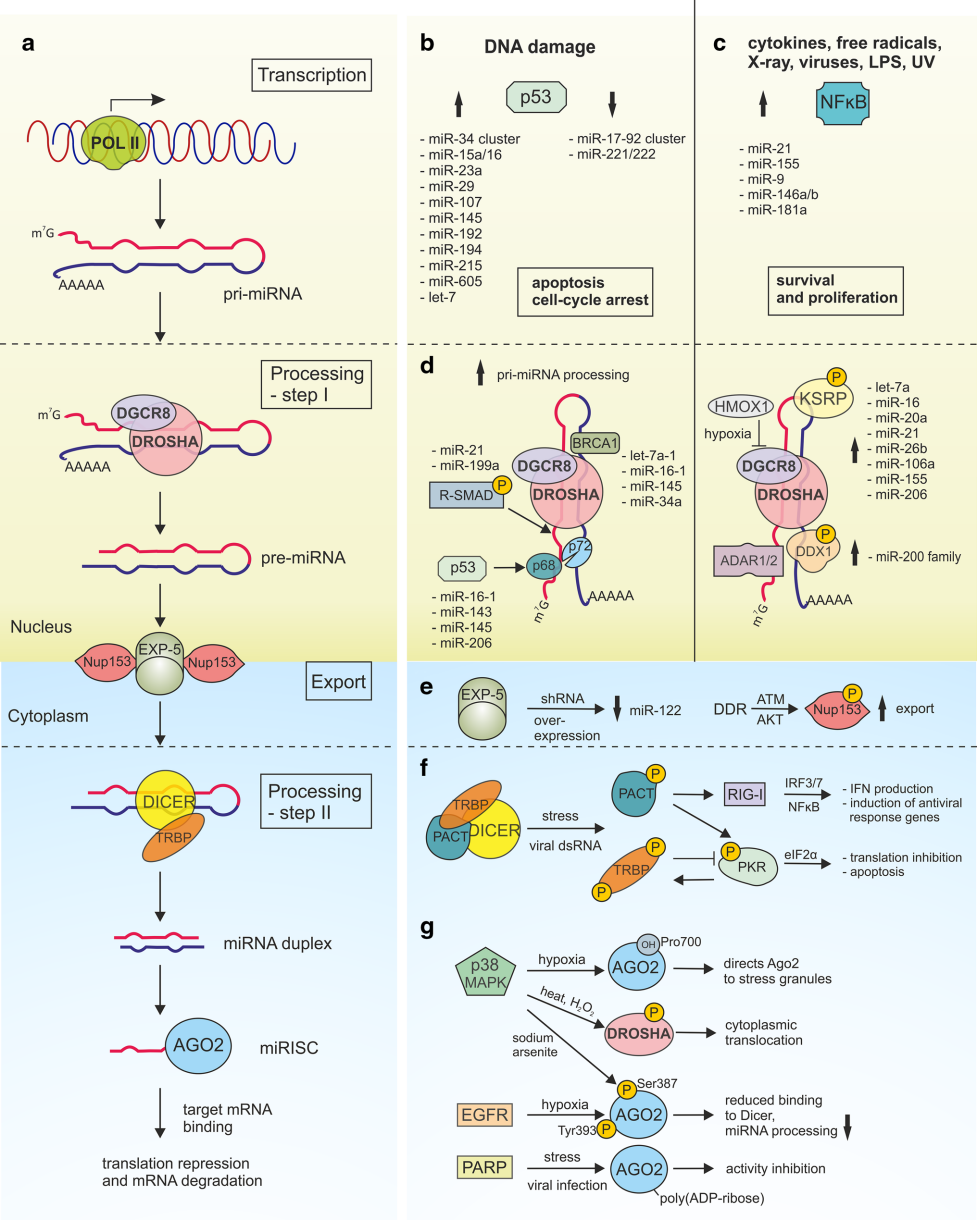
Stress-induced deregulation of miRNA biogenesis. **a** The biogenesis of miRNA begins with RNA polymerase II-dependent transcription that generates pri-miRNA. During the first processing step, pri-miRNAs are cropped to pre-miRNAs by the microprocessor complex composed of Drosha and DGCR8. Next, pre-miRNAs are transported from the nucleus to the cytoplasm by Exportin -5/RanGTP complex where they undergo processing by the RNase III protein—Dicer with the cofactor protein TRBP. Stem-loop structure of pre-miRNA is cleaved to the short miRNA duplex that is approximately 22 nt in length. In the next step, the miRNA is incorporated into the RNA-induced silencing complex (RISC), and following unwinding and strand selection, the mature miRNA can recognize the target sequence that is localized mainly in the 3′ UTRs of transcripts. Binding of the miRISC to the target results in translational repression and/or deadenylation and degradation. **b**, **c** Transcriptional deregulation of miRNA is triggered by various stresses that are responsible for activation of transcription factors such as p53 and NF-κB. **b** Deregulation of miRNAs and their target genes results in cell-cycle arrest and apoptosis (p53-dependent signaling) or **c** influences survival and proliferation (NF-κB-dependent signaling). **d** The first step of miRNA processing is regulated by stress and results in enhanced or suppressed miRNA maturation. Upon stimulation, transcription factors, such as R-SMADs, p53 and BRCA1 facilitate the processing of some pri-miRNAs by direct pri-miRNA binding or stabilization of microprocessor complex. RNA-binding proteins, such as ADAR1, ADAR2, KSRP and DDX1 can also regulate pri-miRNA processing upon stress-conditions. Moreover, pri-miRNA processing may be suppressed during oxidative stress by heme oxygenase-1 (HMOX1)—mediated DGCR8 inhibition. **e** The export of pre-miRNA from the nucleus to the cytosol may be disturbed upon stress conditions. Exportin-5 (EXP-5) saturation by shRNA overexpression results in down-regulation of mature miR-122. Upon DNA damage, ATM-activated AKT kinase phosphorylates nucleopore component Nup153, thus enhancing its interaction with EXP-5 and promoting the export of pre-miRNAs into the cytoplasm. **f** Cross-talk between miRNA biogenesis machinery and the innate immune response. RIG-I and PKR are the cytoplasmic sensors of foreign RNA. Upon foreign dsRNA recognition and activation, PKR blocks translation by the phosphorylation of the eukaryotic translation initiation factor 2α (eIF2α) and induces IFN signaling pathways. TRBP and PACT which form a functional complex with Dicer inhibit and activate PKR, respectively. PACT can also activate RIG-I signaling, leading to IRF3/7 and NF-κB activation, IFN production and antiviral response. **g** The key miRNA biogenesis proteins undergo various post-translational modifications and subcellular translocation in response to stress conditions. Stress regulates Ago2 by its p38/MAPK-dependent phosphorylation at serine 387; hydroxylation at proline 700 and epidermal growth factor (EGFR)-dependent phosphorylation at tyrosine 393, which reduces the binding of Ago2 to Dicer and inhibits the processing of tumor-suppressor-like miRNAs. Heat and H_2_O_2_ stress induce Drosha phosphorylation by p38/MAPK leading to its reduced binding to DGCR8 and cytoplasmic translocation. Ago proteins loss their activity as a result of stress-induced poly(ADP-ribosyl)ation

Cellular biogenesis of miRNAs is a multi-step process including the transcription of miRNA genes, mostly by RNA polymerase II, and generation of long primary transcripts (pri-miRNA) that are capped and polyadenylated [6, 7] (Fig. 1a). In the next step, pri-miRNAs are further processed in the nucleus by the microprocessor complex composed of the RNase III Drosha and its RNA-binding protein component, DiGeorge syndrome critical region 8 (DGCR8). Drosha can also interact with DEAD-box helicase (DDX) proteins: DDX5 (known as p68), DDX17 (known as p72) and DDX1 required for the biogenesis of some miRNAs. The emerging approximately 70 nt in length miRNA precursors, also known as pre-miRNAs, with a characteristic stem-loop structure, are transported from the nucleus by exportin-5 (EXP-5). Further processing in the cytoplasm is carried out by the second RNase III, Dicer and its cofactors TAR RNA-binding protein (TRBP) and the protein activator of PKR (PACT), resulting in the formation of a miRNA duplex. After duplex unwinding by the helicase activity of Dicer, one miRNA strand (guide strand) is packed into the RNA-induced silencing complex (RISC), while the other strand (passenger strand) is rapidly degraded. The miRNA-containing RNA-induced silencing complex (miRISC), with the catalytic component an Argonaute (Ago) protein, recognizes the imperfectly matched complementary sequences in its target, localized mainly in 3′ UTRs, leading to the translational repression and/or accelerated transcript degradation by uncapping and deadenylation. The miRNAs recognize their targets primarily through complementarity within the seed sequence—at nucleotides 2–8 of the 5′ miRNA end.

There are many examples of the non-canonical miRNA biogenesis pathway. For example, some miRNAs are grouped in clusters and are transcribed/processed together; intronic pri-miRNAs (mirtrons) are Drosha-independent and are released by the spliceosome [8]; some miRNAs can be processed independently of Dicer with the use of Ago2, which can cleave the miRNA precursors and produce mature miRNAs; and recently discovered agotrons escape the conventional biogenesis pathway entirely and associate with Ago proteins as full-length introns [9].

The number of steps and protein factors involved in miRNA biogenesis offer numerous possibilities to fine-tune this process to precisely regulate the mature miRNA levels. Perturbation of cellular homeostasis may disrupt each of the miRNA biogenesis steps, leading to deregulation of pathways and complex gene expression networks controlled by miRNAs. As a result, the cell is more prone to stress and its harmful effects. Such a deregulation is observed in a variety of human diseases, including cancers as well as metabolic and cardiovascular diseases. miRNAs can function as oncogenes or tumor suppressors, and generally miRNA down-regulation is observed in tumor cells [10, 11]. It has been demonstrated that altered expression of miRNA in response to stress (radiation) can suppress resistance of cancer cells to therapy [12]. miRNAs are also useful for the classification, diagnosis, prognosis and experimental treatments of diseases [13]. Therapeutic agents and their delivery carriers may also serve as specific types of stressors, which may activate cellular sensors of foreign RNA and DNA or induce DNA damage response (DDR) [14].

Numerous studies have shown that DNA damage stress changes the global profile of miRNA expression [15–17]. The second type of stressors widely studied in the context of miRNA deregulation is pathogen infection and innate immune response activation [18, 19]. In this review, we will focus on stress-induced changes in miRNA transcription, processing and activity. The published data presented here support the hypothesis that stress directly influences not only the miRNA quantity but also the functioning of the core miRNA biogenesis proteins as well as affects the final length and sequence of mature miRNA (isomiRs). We also discuss the role of chronic stress and miRNA deregulation in the context of age-associated neurodegenerative disorders, such as Alzheimer’s disease (AD), Parkinson’s disease (PD), Huntington’s disease (HD) and amyotrophic lateral sclerosis (ALS).

## Modulation of transcription upon stress

Similar to any other gene products, the expression of miRNAs is controlled at the transcriptional level by transcription factors, and in recent years, the knowledge of miRNA promoters and regulators has deepened considerably [20]. Nevertheless, the level of mature miRNA is not simply the result of transcription, but its stability and processing steps also influence the cell-type and stage-specific expression. Even subtle changes in miRNA levels (approximately two-fold) may induce significant physiological effects upon stress conditions by regulating transcription factors [21] and other signaling molecules. There are examples of miRNAs working within positive, negative and double-negative feedback loops, which allows for the regulation of the signal strength and the duration of the stress response [1].

An example of transcriptional regulation of miRNA expression upon stress is DNA damage response, an evolutionarily conserved system to maintain genome integrity in eukaryotes [2]. Genotoxic agents, such as UV radiation, ionizing radiation (IR), oxidative stress and chemical mutagens, can activate transcription factors, including p53, NF-κB, c-Myc, and c-Jun, that up- or down-regulate the expression of specific miRNAs.

### miRNA regulation by p53

A decade ago seven groups reported independently that the transcription factor encoded by the p53 tumor suppressor gene is a key regulator of the miR-34 family genes [22–28]. Under basal conditions, p53 expression level is tightly regulated by its ubiquitin-mediated degradation and repression by miR-125b [29]. A wide range of stresses, including DNA double-strand breaks activate ataxia-telangiectasia mutated (ATM) serine/threonine kinases that in turn phosphorylate p53 to arrest cell cycle for DNA repair [1]. Active p53 induces the transcription of miR-34a and miR-34b/c genes that repress the expression of target genes (such as the transcription factor c-Myc) to promote the induction of apoptosis, cell cycle arrest, and senescence [30]. A highly conserved p53 binding site is located near the transcription start site of miR34a and miR34b/c genes. It was previously proposed that due to high sequence homology, they regulate the same targets, mainly cell cycle-regulatory genes [22, 25]; however, a recent study suggests that miR-34a and miR-34b/c might serve different biological functions [31]. According to the miRTarBase, hsa-miR-34a has 670 targets, and more than 100 of these were validated with the use of two or more methods [32]. Some of these targets include are cyclin dependent kinases (CDK4/6), cyclin E2 (CCNE2), BCL2, MYC, MET and E2F transcription factor 5 (E2F5). The introduction of miR-34a into healthy or tumor cells induces senescence and apoptosis, whereas the inhibition of miR-34a with the use of antimiR-LNA reduces DNA-damaged induced apoptosis [26].

Morover, p53 can transcriptionally activate other miRNAs with antiproliferative activities (such as, miR-15a/16, miR-23a, miR-29, miR-107, miR-145, miR-192, miR-194, miR-215, miR-605, let-7) and transcriptionally repress miR-17-92 cluster and miR-221/222 [30] (Fig. 1b). For example, miR-15a/16, miR-29, miR-192, miR-215 and let7 target the oncogene BCL2 regulating DNA damage-induced apoptosis [27]. In addition, miR‐29 participates in a negative feedback loop by down-regulation of its targets that are negative regulators of p53 (CDC42 and the p85α-regulatory subunit of phosphoinositide-3Kinase PI-3K) [33]. P53/PI-3K/Akt -mediated activation of miR-145 reduces the expression of proto-oncogene c-Myc, resulting in the inhibition of tumor cell growth both in vitro and in vivo [34]. Interestingly, c-Myc-mediated transcriptional effects for many miRNAs are opposite to those regulated by p53. The miR-34 family of genes is repressed by a c-Myc similar to miR-15a/16, miR-23a, and miR-29. However, p53- repressed miRNAs such as miR-17-92 and mi221/222 are activated by c-Myc.

### miRNA regulation by NF-κB

The transcription factor NF-kappa-B that is expressed in almost all cell types plays a crucial role in cell proliferation, differentiation, immunity, inflammation, and stress response through regulating the expression of a variety of genes. It responds to a broad range of different stimuli, including cytokines, UV and X-ray, free radicals, viruses (proteins, dsRNA) and bacterial lipopolysaccharide (LPS). The aberrant activation of NF-κB has been linked to inflammatory and autoimmune disorders as well as cancer. Generally, p53 and NF-κB have opposing effects in cells (apoptosis and cell cycle arrest for p53 versus survival and proliferation for NF-κB), and therefore, cannot function at the same time [35].

A direct link between stress-induced NF-κB activation and the up-regulation of some miRNAs, including miR-21, miR-155, miR-9, and miR-146a/b, has been reported [36] (Fig. 1c). The first example, the oncogenic miR-21, is deregulated in a significant number of diseases including solid tumors. Genotoxic agents up-regulate miRNA-21 expression by recruiting NF-κB and signal transducer and the activator of transcription 3 (STAT3) to the miR-21 promoter region. This cooperative action is mediated by the NF-κB-dependent IL-6 up-regulation that is responsible for STAT3 activation upon genotoxic stress [37]. A complex relationship between miR-21 and NF-κB has been reported [38]. Depending on the cell type, miR-21 may positively or negatively regulate NF-κB; for example, in epithelial cells, miR-21 acts to down-regulate phosphatase and tensin homolog (PTEN), activate protein kinase B (known as AKT), and increase NF-κB activation promoting survival and growth; in LPS-stimulated macrophages, miR-21 works to inhibit NF-κB and its proinflammatory signaling [39, 40].

Upon inflammatory stimuli (for example, Toll-like receptor ligands and proinflammatory cytokines) in immune cells, NF-κB up-regulates the transcription of inflammatory-responsive genes as well as miR-9, miR-155 and miR-146a/b, which down-regulate the proinflammatory signaling cascade in a negative feedback loop. For example, NF-κB-mediated miR-146a production is rapidly up-regulated after the exposure of myeloid and T cells to LPS, tumor necrosis factor alpha (TNF-α) and interleukin 1 beta (IL-1β). This results in the silencing of TNF receptor associated factor 6 (TRAF6) and interleukin 1 receptor associated kinase 1 (IRAK1), which are NF-κB activators. Interestingly, mature miRNAs accumulate later than immediately transcribed pri-miRNAs, and this delay acts as the timer for stress response during inflammation. MiR-146a is also up-regulated during vesicular stomatitis virus (VSV) infection in mouse macrophages. This effect depends on the viral RNA recognition by the cytoplasmic sensor retinoic acid-inducible gene I (RIG-I) and the subsequent NF-κB activation. Through targeting TRAF6, IRAK1 and IRAK2, miR-146a negatively regulates VSV-triggered type I IFN production and antiviral response [41].

The final regulatory effect of miRNA is a result of the relative concentration of miRNA and its targets; therefore, the stress-induced up- or down-regulation of one component influences the other. This interplay results in a specific timing of target gene expression. The important feature of these networks is that individual miRNA regulates multiple targets, and transcription factors become key activators of these pathways by regulating both miRNAs and their targets. Moreover, one mRNA target may be under the control of multiple miRNAs that may bind cooperatively [42, 43]. Such a complex regulatory system allows for a rapid stress response, signal strength regulation and restoration of cellular homeostasis.

## miRNA biogenesis and stress

The direct comparison of pri-miRNA, pre-miRNA and mature miRNA forms revealed that in some cases, their amounts were not correlated. Growing evidence indicates that stress regulates not only transcription but also the biogenesis of miRNAs [18, 30, 44–46]. Different stressors may induce the accumulation of pre-miRNAs and the reduction of mature miRNAs, or they can facilitate the processing of some miRNAs. In addition to expanding our knowledge on miRNA biogenesis, numerous factors working within miRNA biogenesis complexes and the co-regulators of miRNA precursor maturation were identified (Fig. 1d–g). The vast majority of examples describing various regulatory effects applies to the microprocessor step of miRNA biogenesis and transcription factors, including SMADs, p53 and breast cancer 1 (BRCA1), which are key players in this process (Fig. 1d).

The first transcription factors that were found to regulate miRNA biogenesis are SMAD proteins [47]. The Hata group demonstrated that SMADs transduce signals from the transforming growth factor-β (TGF-β) and the bone morphogenetic protein 4 (BMP4) and mediate the rapid, posttranscriptional induction of miR-21 and miR-199a in human primary pulmonary smooth muscle cells. Upon stimulation, the Receptor-specific SMADs (R-SMADs) undergo phosphorylation, translocate to the nucleus, associate with the Drosha/DGCR8/p68 microprocessor complex, and facilitate the cleavage of pri-miRNA to pre-miRNA by Drosha. SMADs recognize specific sequence motifs within the pri-miRNA stem region (5′-CAGAC-3′), and the mutation of this sequence abrogates the ligand-induced recruitment of the microprocessor complex and processing [48]. There are approximately 20 miRNAs that are regulated post-transcriptionally by ligand-dependent induction of TGF-β/BMP and SMAD proteins.

In addition to its role in transcriptional gene regulation, p53 was shown to post-transcriptionally regulate the expression of various miRNAs, including miR-16-1, miR-143, miR-145 and miR-206 [30]. It has been demonstrated that after the stimulation of the human colon cancer cell line (HCT116) with DNA-damaging agent doxorubicin, some miRNAs show increased expression of their pre- and mature forms in contrast to primary transcripts, which remained unchanged. This effect was p53- and p68/p72-dependent. As a result of DDR, p53 interacts with the microprocessor complex through direct binding to p68 and enhances the Drosha-mediated processing of several miRNAs (Fig. 1d). Through targeting cell cycle and proliferation pathways, miRNAs support the growth-suppressive function of p53 upon stress conditions. This regulatory network is further complicated by the fact that tumor suppressor BRCA1 can facilitate the processing of pri-let-7a-1, pri-miR-16-1, pri-miR-145, and pri-miR-34a by direct RNA binding and association with the Drosha microprocessor complex and SMADs/p53 [49]. BRCA1 is activated by ATM after DNA damage and plays a crucial role in DNA repair.

The step of pri-miRNA to pre-miRNA processing is also controlled by single-stranded RNA- binding KH-type splicing regulatory protein (KSRP), a key component of both Drosha and Dicer miRNA-processing complexes [50]. Trabucchi et al. demonstrated that KSRP selectively binds to the terminal loop of some pri-miRNAs, with preference to short G-rich stretches, and facilitates their processing [50]. KSRP knock-down in both HeLa and NIH-3T3 cells revealed that the levels of mature let-7a, mir-16, miR-20a, miR-21, miR-26b and miR-106a were reduced by 40–70%. Further studies identified a direct interaction between KSRP and ATM kinase [51]. Upon DNA damage, the ATM kinase directly phosphorylates KSRP and facilitates its function in miRNA maturation. A similar regulatory pathway is demonstrated for the double-stranded RNA-binding protein DDX1, which also promotes the maturation of a subset of miRNAs (such as the miR-200 family), and the majority of them are induced by DNA damage [52]. Phosphorylation of DDX1 by stress-induced ATM kinase enhances its binding to pri-miRNAs and recruiting the Drosha complex [52].

During C2C12 myoblast differentiation PI3 K/AKT kinase signaling activation induces the expression of myomiRs (e.g., miR-206) by KSRP-dependent facilitation of pri-miRNA to pre-miRNA processing [53]. Phosphorylation of KSRP by AKT inhibits its ability to bind with mRNA and promotes the pri-miRNA-binding. A similar effect was also observed in the immune system [54]. LPS is a glycolipid of the outer cell membrane of Gram-negative bacteria. It is recognized by the transmembrane protein toll like receptor 4 (TLR4) that activates various transcription factors, including NF-κB, leading to the increased production of proinflammatory cytokines, such as TNF-α and IL-6. In macrophages, LPS significantly induces the expression of miR-155, and this induction depends on KSRP-mediated enhanced maturation of its precursor [54].

Another example of a protein involved in miRNA biogenesis is hnRNP A1, which by binding to the loop of pri-miR-18a promotes its cleavage by Drosha [55]. Interestingly, it has an antagonistic effect to KSRP during the let-7a biogenesis [56].

It has been shown that key miRNA biogenesis proteins, such as Drosha, Dicer and Ago2, are down-regulated by hypoxia [57–59]. Upon oxidative stress, a total pool of pre-miRNA and miRNA in myoblasts is decreased through DGCR8 regulation by heme oxygenase-1 (HMOX1) [60] (Fig. 1d). Heme is essential for DGCR8 activity [61], and the heme-binding domain of DGCR8 plays a key role in pri-miRNA recognition [62]. Hypoxic stress also regulates Ago2 by its phosphorylation (EGFR-dependent and p38/MAPK-dependent) [63, 64] and hydroxylation [65], which reduces the binding of Ago2 to Dicer or directs Ago2 to stress granules [66], respectively. Ago function is also regulated by stress-induced poly(ADP-ribosylation) [67, 68] (Fig. 1g).

## Interferon regulation of miRNA biogenesis and functioning

In mammals, the innate immune response plays a key role in response to stress induced by pathogen-associated molecular patterns (PAMPs). The signals derived from microorganisms include unmethylated CpG DNA, double-stranded RNA, 5′-triphosphate RNA and LPS. PAMPs are recognized by cells via conserved sensors, known as pattern-recognition receptors (PRRs), such as Toll-like receptors, an IFN-inducible dsRNA-activated protein kinase (PKR) and retinoic acid-inducible gene I (RIG-I)—like receptors (RLRs). After stimulation, PRRs induce intracellular signaling pathways, involving transcription factors [e.g., NF-B, activator protein 1 (AP-1), IFN regulatory factors (IRFs)], and the synthesis of signaling molecules, such as cytokines, chemokines, and immunoreceptors. IFN production subsequently results in the up-regulation of approximately 2000 IFN-stimulated genes (ISGs) in an IFN subtype-, dose-, cell-type- and stimulus-dependent manner [69]. There are more than 30 known miRNAs regulated by type I IFNs, including those regulated by IFNβ (miR-155, miR-129 and miR-196a), IFNα (for example, miR-143 and miR-378), and both IFNβ and IFNα in a cell type-specific manner (such as the let-7 family and miR-30) [19]. IFNs up-regulate miRNAs with antiviral activity (such as miR-29 and miR-196b). For example, the expression of miR-196b and eight other miRNAs predicted to target HCV RNA is modulated upon type-I IFN treatment of primary mouse hepatocytes, Huh7 cells [70] and human peripheral blood mononuclear cells (PBMC) [36]. Contrarily, the antiviral effects were also observed by the down-regulation of liver-specific miR-122 in response to IFNβ [70, 71].

Moreover, many core miRNA biogenesis proteins have also been implicated in the IFN response. Down-regulation of Ago1 and Ago2 [19, 69] was observed in lung and blood cells 24 h post-IFNα stimulation. Dicer down-regulation was observed 72 h after IFNα and poly(I:C) treatment in JAR and HeLa cells as well as in mice and human tissues [72]. This effect was not correlated with apoptosis. Furthermore, oxidative stress selectively inhibits Dicer but not Ago1 in JAR cells. Contrastingly, IFN-γ (type II class of IFNs) up-regulates Dicer in JAR and HeLa cells [72].

A well-known example of the link between miRNA biogenesis machinery and the innate immune response is TRBP/PACT and PKR cross-talk [73–77] (Fig. 1f). Knocking down TRBP and PACT influences miRNA biogenesis; however, there are some differences between studies performed in vitro and in cells [78–80]. TRBP controls mature miRNA length and strand selection [81]. The depletion of TRBP in vitro and in mouse and fly systems results in the lower accuracy of Dicer cleavage, leading to the generation of shorter iso-miRs [82, 83]. TRBP and PACT, both of which form a functional complex with Dicer [74, 83], have been reported to inhibit and activate PKR, respectively. Upon foreign dsRNA recognition and activation, PKR blocks translation by the phosphorylation of a eukaryotic translation initiation factor 2α (eIF2α) and induces IFN signaling pathways. A transient blockade in translation directs cellular metabolism toward damage repair. TRBP is hyperphosphorylated by c-Jun N-terminal kinase (JNK) when PKR is activated by dsRNA, and this modification enhances the inhibitory activity of TRBP on PKR [84]. It has been demonstrated that during viral infection PACT directly binds to the other cytoplasmic sensor of foreign RNA, RIG-I and stimulates its ATPase activity to trigger innate antiviral response [85].

miRNA biogenesis and regulatory networks may be disturbed by stressors in the form of exogenous RNA and DNA (such as plasmid DNA, antisense oligonucleotides, RNA interference tools, antimiRs or miRNA sponges) used in various experimental approaches, including therapy [14, 86, 87]. It has been recently reported that a genetic tool Cre/loxP-based recombination system may induce DNA damage and accumulation of cytoplasmic DNA products that are sensed by the cytoplasmic sensor of DNA—STING [88]. Thus, strong type-I IFN antiviral response in different mouse and human cell models was observed. Since a wide range of genes may be stimulated by IFNs, we speculate that it may influence miRNA biogenesis and functional networks; however this hypothesis remains to be corroborated. A deeper insight into similar problems is also required in the context of genome editing technology.

## Changes in miRNA/protein complex localization upon stress

The export of pre-miRNA from the nucleus is another key step of miRNA biogenesis that undergoes regulation and may determine the level of active miRNA in the cytoplasm. Pre-miRNAs are transported by EXP-5 (encoded by *XPO5*), a Ran guanosine triphosphate (RanGTP)–dependent dsRNA-binding receptor [89]. There are only a few reports describing the role of stress in the regulation of pre-miRNA transport. More than a decade ago, the Kay group reported that the overexpression of artificial pre-miRNAs (short hairpin RNAs) in the liver of adult mice saturates the miRNA biogenesis pathway at the EXP-5 step [90]. This leads to the high toxicity of reagents in vivo and the down-regulation of liver-derived miR-122 (Fig. 1e).

The opposite effect was observed upon DNA damage, when ATM-activated AKT kinase phosphorylates Nup153, an important nucleopore component, thus enhancing its interaction with EXP-5 and promoting the export of pre-miRNAs into the cytoplasm [91] (Fig. 1e). Another export protein that facilitates the transport of RNAs and proteins across the nuclear membrane to the cytoplasm is Exportin-1 (EXP1, also known as the Chromosomal Maintenance 1). Some non-canonical pre-miRNAs (e.g., pre-miR-320) are transported by EXP1 [92]. Interestingly, a broad range of viral PAMPs, including dsRNA, trigger EXP1-mediated Drosha translocation from the nucleus to the cytoplasm in murine fibroblasts [93]. Heat and H_2_O_2_ stress might induce Drosha phosphorylation by p38 MAPK, leading to its cytoplasmic translocation, which was responsible for the reduced binding of DGCR8 by Drosha and its destabilization and degradation by calpain [94] (Fig. 1g).

Under stress, cell metabolism is diverted toward survival and eventual recovery. One way to conserve energy is to limit the translation and focus only on producing proteins needed for survival. In eukaryotic cells, non-translating mRNAs and their associated RNA-binding proteins aggregate into structures called ribonucleoprotein (RNP) granules. They form within minutes in response to stress to protect cellular mRNAs. Subcellular structures known as processing bodies (PBs) are the major sites of RNA processing and degradation in most cells [95]. During the stress-induced translational arrest, stalled initiation complexes and mRNAs with RNA-binding proteins assemble into stress granules (SG) [44, 96, 97]. miRNAs and proteins involved in miRNA biogenesis have also been shown to associate with SG following the onset of cell stress. For example, arsenite treatment triggers Ago2 relocalization into stress granules [98]. Moreover, oxidative stressors enhance the interactions of many proteins, including Ago2 and SG components, with Dicer, resulting in its inhibited catalytic activity [45]. UV irradiation and H_2_O_2_ also induced SG formation but only in a cell cycle-dependent mode [16]. The accumulation of cytoplasmic granules is a known hallmark of neurodegenerative disorders and will be described in the last section.

## miRNA modifications in response to stress

Stress-induced post-transcriptional modifications of miRNAs include changes in the miRNA sequence, modifications of miRNA ends and “strand switching.” Inflammation-induced adenosine deaminase acting on RNA (ADAR) up-regulation can influence pri-miRNA processing [99]. Conversion of adenosine to inosine (recognized as guanosine) is the most prevalent form of RNA modification in higher eukaryotes. Modified transcripts are recognized by the cellular sensor of dsRNA (MDA5) as self versus non-self viral RNAs [100]. The A-to-I editing can alter the structure of pri-miRNAs, which may result in (1) miRNA processing inhibition [99, 101, 102] or (2) activation [103], (3) new mRNA targets recognition and (4) RISC loading suppression. For example, the small RNA-seq data from a hypoxia-treated breast cancer cell line identified 31 statistically significant modification sites in 21 different miRNAs. Most A-to-G modifications occurred in seed regions, whereas the levels of miRNA modifications generally increased with the time of exposure to stress. These modifications may have a direct influence on targeting, as shown for miR-27a-3p [104]. Expression profiling of small RNAs from brain tissues showed that more than 80% of miRNA is heterogeneous in length, and 3′-trimming variants were the most predominant [46]. Among isomiRs in the seed region, A–G changes were frequent at position 5 (miR-411 and miR-379), position 6 (miR-376 cluster) and several other positions (miR-320) [46]. Regardless of the catalytic activity, ADAR1 can also form a functional complex with Dicer and Ago2. This protein–protein interaction is responsible for the induction of conformational changes and increasing the rate of pre-miRNA processing by Dicer, RISC assembly, and loading of miRNA [105].

Pre-miRNAs and miRNAs are often heterogeneous at their 3′ and 5′ ends as a result of imprecise Drosha and/or Dicer cleavage and tailing (non-templated nucleotidyl addition to the 3′ end of RNA) or trimming activity [46, 106]. Despite the fact that most miRNAs are 3′ tailed or 3′ trimmed, the biological significance of isomiRs in most cases remains unknown. The 5′ end variants with changed seed sequence may regulate different sets of target genes. However, the 3′ end modifications (adenylation and deadenylation) have been associated with specific functional consequences [107, 108]. After transport into the cytoplasm, some pre-miRNAs are modified by uridylation, which is one of the most frequent types of RNA tailing [109]. RNA tailing is carried out by terminal uridylyl transferases (TUTases) that recognize the overhang of a pre-miRNA. For example, TUT4 recruited by Lin-28, mediates the uridylation of pre-let-7 miRNA [110], thus preventing the further processing of pre-let-7 miRNA and inhibiting its function.

Recently, we demonstrated that plasmid-based RNA interference reagents, empty vectors and poly(I:C) induce changes in the composition of 3′ miR-221/222 isomiRs in human fibroblasts [87]. As the transfection of all these reagent types induces apoptosis, miR-221/222 shortening may be part of this process. The mechanism of these 3′ isomiRs formation and the role of shorter miRNA variants is unknown; however, it may result from altered pri-miRNA processing by Drosha or stress-induced miRNA trimming. The length of the 3′-terminus of a miRNA may modulate its interaction with the target, and therefore, it may influence miRNA stability [111]. We speculate that stress conditions may also change the relative ratio of canonical vs. non-canonical targets of miR-221/222, thus stabilizing some isomiR variants.

Microbial infections influence miRNA repertoire [112]. For example, the miR-15 family is down-regulated during Salmonella infection through the inhibition of the transcription factor E2F1 [113]. Virulent mycobacteria induce the expression of the miR-132/212 family [18]. Infection can alter the relative expression of the miRNA arms (arm switching), as it was shown for miR-361 and miR-582 following *Yersinia pseudotuberculosis* infection [18]. Bacterial infection can also change the isomiR distribution in infected cells with the most predominant form of 3′ isomiRs. Interestingly, seven miRNAs showed a change in the 5′ end, thus altering a seed sequence [18].

As we described earlier, miR-34 is regulated in cells by p53 during DNA damage response. Salzman et al. demonstrated that there is a pool of inactive mature miR-34 in cancer cell lines [114]. Radiation-induced ATM/hClp1 kinase activates this pool by phosphorylation of miR-34 at the 5′ end. This mechanism, independent of the de novo transcription and processing, represents the rapid response of the cell to environmental stimuli.

## Chronic stress—miRNA in neurodegeneration

Over the past decade, miRNAs have emerged as important regulators of aging and neurodegeneration. Neurodegenerative diseases are a group of late-onset progressive disorders resulting from the increased accumulation of toxic proteins and leading to neuronal dysfunction. The disease progression may be further modulated by aging and environmental factors such as infections or exposure to toxins. Moreover, the role of inflammation in neurodegeneration is well documented [115, 116]. Because harmful molecules accumulate over time in non-dividing neurons, they may act as chronic stressors. The best-studied neurodegenerative diseases are PD, AD, HD and ALS. The role of individual miRNAs in the pathogenesis of these diseases and their potential as biomarkers and therapy targets has been intensively studied [117–122]. Many deregulated miRNAs have been reported in cell lines, patients’ tissues and animal models of neurodegenerative disorders (Supplementary Table 1). However, it is not clear if this is a cause or a consequence of the disease. Some deregulated miRNAs are common for different disease models (such as miR-9, miR-29, miR-34a/b/c, miR-125, miR-128, miR-132 and miR-424) [118, 122–138], whereas others are disease-specific [139–147]. Here, we characterize toxic proteins specific for selected diseases and present a few examples of miRNA deregulation in neurodegenerative disorders.

The classic pathological feature of PD is the loss of midbrain dopaminergic neurons (DNs) of the substantia nigra and the presence of cytoplasmic inclusions (called the Lewy bodies) in the remaining neurons [148]. The maturation and functioning of the DNs are regulated by miR-133b that works within a negative feedback loop with paired-like homeodomain transcription factor Pitx3 [149, 150]. Expression analysis confirmed by RNase protection assays and northern blotting revealed that miR-133b (both pre- and mature miRNA) was specifically absent in PD patient samples and the midbrain of Pitx3 mutant Aphakia mice [149]. The transcription factor Pitx3 is a direct target of miR-133b, and the overexpression of Pitx3 leads to the up-regulation of pre-miR-133b. Other miRNAs deregulated in PD include miR-7, miR-10b, miR-34b/c, miR-132, miR-135a and miR-433 [132, 138–140, 151–153].

Common neuropathological features of AD cases are β-amyloid neuritic plaques accumulation and intraneuronal neurofibrillary tangles (containing tau protein). Beta secretase 1 protein (BACE1) is responsible for the cleavage of amyloid precursor protein (APP) and amyloid β production. Alpha-synuclein (α-syn) insoluble fibrils are found in both sporadic and familial cases with AD and rare Mendelian forms of PD. APP and BACE1 are targeted by numerous miRNAs, including miR-9, miR-16, miR-106b, miR-107, miR-124, miR-153, miR-195 and miR-29, which are down-regulated in AD patients (Supplementary Table 1) [119, 154–161]. In some cases, the miRNA deregulation was AD grade-dependent. For example, miR-16 and miR-146a were up-regulated in early AD cases (Braak III/IV) and down-regulated in hippocampal samples from the late stages of AD (Braak VI). Lower levels of miR-146a were also observed in the cerebrospinal fluid (CSF) of AD cases [119].

Elevated levels of miR-34c were observed in the circulating blood plasma [162], the hippocampus of AD patients and the corresponding mouse models [133]. miR-34c inhibitors reinstated learning behavior and physiological SIRT1 levels (miR-34c target), indicating that miR-34c directly contributes to AD and age-associated memory impairment. Contrastingly, the expression profiling of PD brain samples revealed that miR-34b/c are down-regulated at early (pre-motor) and late (motor) stages of the disease. The authors suggested that the depletion of miR-34b/c leads to mitochondrial dysfunction, oxidative stress, and a decrease in the expression of DJ1 and Parkin associated with familial forms of PD [132]. Further inhibition of miR-34b/c in SH-SY5Y cells increased α-syn levels and stimulated aggregate formation, thus confirming the role of the miR-34 family in neurodegeneration [163].

HD is the best-known example of a group of dominantly inherited neurological diseases caused by the expansion of unstable CAG repeats in coding regions of the associated genes. This group also includes spinocerebellar ataxia type 1, 2, 3, 6, 7 and 17 (SCA), spinobulbar muscular atrophy (SBMA) and dentatorubral-pallidoluysian atrophy (DRPLA). Toxic polyglutamine (polyQ)-rich proteins form intracellular aggregates that affect numerous cellular activities leading to neurodegeneration. Moreover, mutant transcripts may also play an important role in neurodegeneration, as it was shown for untranslated trinucleotide diseases such as myotonic dystrophy type 1 (DM1). The common feature of various cell lines modeling polyQ diseases is the formation of nuclear RNA foci containing transcripts with an expanded CAG tract. Strong miRNA and isomiR expression deregulation were observed in the frontal cortex and the striatum of HD patients [46]. In the group of deregulated miRNAs, p53 and RE1 silencing transcription factor (REST) targets were enriched, suggesting the role of these transcription factors in HD pathogenesis. Gaughwin et al. demonstrated by qPCR assay that p53-regulated miR-34b is up-regulated in response to mHTT in both pluripotent and neuronally differentiated human cells and in human plasma. Interestingly, significantly elevated levels of miR-34b were observed in plasma from the HD gene carriers before symptom onset and therefore may be used as a biomarker for HD [118]. Massive parallel sequencing of small RNA libraries revealed that miR-34c is highly enriched in the hippocampus, in contrast to the miR-34b, which is transcribed from the same cluster [133].

MiR-9 and miR-124a regulate neuronal proliferation and differentiation. As a key regulator of microglia quiescence in the CNS, miR-124a helps in preventing CNS inflammation [164]. Such abundant miRNAs in the brain are regulated by the REST—inhibitor of neuronal gene expression in a non-neuronal cell. Mir-9/9* is decreased early in HD and targets REST and corepressor for REST (CoREST), respectively, forming double negative feedback loop [123]. Interestingly, unchanged pre-miR-124a [126] levels in contrast to reduced mature miRNA levels were observed in the HD cortex [123], implying post-transcriptional regulation. Decreased levels of miR-124 were also found in AD brains, suggesting the role of miR-124 in pathology of AD and HD.

ALS is caused by the mutations in protein-coding genes such as TDP-43, FUS and hnRNPA1. These RNA-binding proteins contain prion-like domains that allow them to rapidly self-associate and form RNP granules in the cytoplasm of motor neurons. At physiological conditions, TDP-43 and FUS are nuclear proteins that can be reversibly shuttled to the cytoplasm upon stress, where they associate with SGs. This shuttling is dysregulated in ALS; however, it is unclear if this mislocalization and SG aggregation is a cause or consequence of the disease [165, 166]. Interestingly, TDP-43 is a component of the Drosha and Dicer complexes and promotes microRNA biogenesis in the nucleus and cytoplasm [167]. It has been demonstrated that the disruption of the miRNA biogenesis pathway by Dicer silencing causes degeneration of various neural cell types in culture and in vivo [168]. Decreased Dicer catalytic activity resulting in global miRNA down-regulation was observed in the motor neurons of ALS patients [45]. The authors suggest that stress and SG formation are responsible for the dynamic changes in Dicer interactions with protein factors, including Ago2 leading to deregulation of miRNA processing.

## Conclusion

Organisms are constantly exposed to various stresses throughout their lives. Some of them induce temporal changes in cellular homeostasis, whereas others deregulate the system permanently, leading to cancers, neurodegeneration or pathogen infections. miRNAs are well-known guardians of cellular homeostasis and, together with transcription factors and protein partners, regulate stress responses. In this review, we have presented only the basic information regarding the vast number and variety of potential stressors, miRNAs, and their regulatory networks. Expression profiling methods will help in identifying up- and down-regulated miRNAs upon stress; however, less is known about the mechanisms underlying these effects. Our knowledge about the post-transcriptional regulation of miRNA biogenesis and functioning under stress conditions is still in its infancy. The role of isomiR variants and the interaction of miRNAs with non-canonical targets is an important and currently unrecognized topic in the context of stress and apoptosis. Finally, a better understanding of pathways and mechanisms leading to miRNA dysregulation in aging and neurodegeneration will assist in the determination of new biomarkers for disease and potential therapeutic targets.

### Electronic supplementary material

Below is the link to the electronic supplementary material.

**Supplementary Table** **1** Examples of miRNA deregulation in HD, AD, PD and ALS. (XLSX 28 kb)
